# Correction: Yuldashev et al. Parking Lot Occupancy Detection with Improved MobileNetV3. *Sensors* 2023, *23*, 7642

**DOI:** 10.3390/s24165236

**Published:** 2024-08-13

**Authors:** Yusufbek Yuldashev, Mukhriddin Mukhiddinov, Akmalbek Bobomirzaevich Abdusalomov, Rashid Nasimov, Jinsoo Cho

**Affiliations:** 1 Department of Computer Engineering, Gachon University, Seongnam-si 13120, Republic of Korea; yusufbek02106@gmail.com (Y.Y.); mukhiddinov18@gachon.ac.kr (M.M.); bobomirzaevich@gmail.com (A.B.A.); 2 Department of Artificial Intelligence, Tashkent State University of Economics, Tashkent 100066, Uzbekistan; rashid.nasimov@tsue.uz

## Error in Figures and Figure Legends

1. In the original publication [1], the correct Figure 6 appears below:

2. Figures 7–9 are removed. Figure 10 is updated as Figure 7, below:

3. Figure 11 is updated as Figure 8, below:

4. Figure 12 is reordered as Figure 9. Figure 13 is reordered as Figure 10. Figure 14 is reordered as Figure 11. Figure 15 is reordered as Figure 12. Figure 16 is reordered as Figure 13. Figure 17 is reordered as Figure 14.

## Text Correction

1. In Paragraph 4 of Section 2.1, “Df2×M×Dk2” should be “Df2 × M × Dk2”; “M×N×Df2” should be “M × N × Df2”. In Paragraph 8 of Section 2.1, “feedforward” should be “feed-forward”.

2. The Section titles of 2.2.1 and 2.2.2 are removed. The correct paragraphs of Section 2.2 appear below:

Figure 6 illustrates two different states in which a parking spot can be: occupied or vacant. So, the parking space classification task can be designed as a binary classification task. The parking space classification task can be done via either traditional machine learning approaches or deep learning approaches. The initial image used as the input for the parking lot occupancy detection system is captured using a camera and consists of the entire parking lot. Before starting the classification process, individual parking spots are extracted from the whole parking lot image with provided parking spot locations.

With traditional machine learning approaches, we extract useful image features with different image preprocessing techniques, like histogram equalization or thresholding. After feature vector extraction, we train one of many classification algorithms, like a support vector machine or multilayer perceptron, with the feature vector and their ground truth labels. Various studies have proposed approaches that use feature extraction to classify individual parking spots.

Al-Kharusi et al. [11] introduced an intelligent parking management system exclusively reliant on conventional image processing methodologies. This system encompasses a series of operations including color space conversion, morphological operations (specifically dilation and erosion), thresholding techniques, edge detection algorithms, and a Hough transform. Ahrnbom et al. [12] devised a parking slot occupancy classifier by integrating Integral Channel Features with either Logistic Regression or Support Vector Machine. Initially, ten feature channels were extracted per input image, encompassing elements such as color channels in the LUV color space, gradient magnitude, and quantized gradient channels. Subsequently, feature vectors were efficiently computed from specific feature channels using the integral image approach. Finally, both logistic regression and support vector machine classifiers were trained and tested using the PKLot dataset. Furthermore, in de Almeida et al. [8], the authors not only provided a dataset but also tackled the issue by employing machine learning methodologies. They utilized their dataset, comprising around 700,000 images of parking spaces from multiple cameras in parking lots, to train Support Vector Machine (SVM) classifiers on diverse textural characteristics, including Local Binary Patterns (LBP), Local Phase Quantization (LPQ), and their derivatives. Additionally, they improved detection accuracy by employing combinations of SVMs and employing basic aggregation techniques, such as maximum or average, on the confidence scores generated by the classifiers.

Numerous scholars have acknowledged the constrained adaptability of handcrafted visual features, such as SIFT, SURF, and ORB, to effectively accommodate the intricacies of object appearance variations, which often exhibit high non-linearity, temporal fluctuations, and complexity. Intriguingly, pre-trained Convolutional Neural Networks (CNNs) have demonstrated remarkable efficacy as "off-the-shelf" feature extractors across a diverse array of visual recognition tasks, as evidenced by findings from Razavian et al. [13]. It should be noted that feature engineering is not applied in DL models because these models aim to discover how parking spots are represented, and the classifier is typically integrated into the DL model. Amato et al. [7] developed mAlexNet, a deep neural network tailored for parking occupancy classification. Through comprehensive evaluations on the PKLot and CNRPark-EXT datasets, mAlexNet demonstrates superior performance over AlexNet and LPQ by de Almeida et al. [8] in terms of both classification accuracy and area under the curve (AUC). Notably, despite being significantly smaller in size—approximately three orders of magnitude compared to the original AlexNet [14]—mAlexNet remains feasible for implementation on embedded platforms such as the Raspberry Pi 2 Model B. Nguyen et al. [15] proposed a modified version of mAlexNet along with their own dataset, HUSTPark, taken from two parking fields at the HUST campus. Their model was very compatible and small in size, but that came in the cost of reducing the accuracy of the model compared to original mAlexNet model.

In Nurullayev and Lee’s study [16], they introduced CarNet, a deep neural network (DNN) utilizing dilated convolutional neural networks to assess parking space occupancy status. CarNet takes as input a 54 × 32 RGB image representing a parking slot. Their experiments demonstrate CarNet’s superior performance compared to AlexNet [14] and other established deep learning architectures on the PKLot dataset. Furthermore, CarNet outperforms mAlexNet [7] on the CNRPark-EXT dataset. Despite achieving high precision and robustness, CarNet necessitates manual cropping of parking slot images from the overall parking lot input image. A detailed comparison between CarNet and our proposed approach is presented in the results section. 

In their paper, Xiao et al. [17] address the problem of whether a free parking spot is compatible with the mission of the ego vehicle by tackling parking spot classification based on the surround view camera system. The authors adapt the YOLOv4 object detection neural network, enhancing it with a novel polygon bounding box model suitable for various shaped parking spaces, including slanted parking slots. Notably, this study represents the first detailed investigation of parking spot detection and classification using fisheye cameras for auto valet parking scenarios. The proposed classification approach effectively distinguishes between regular, electric vehicle, and handicap parking spots. By considering both occupancy and suitability, this research contributes to more intelligent and context-aware parking guidance systems. Grbich et al. [18] introduced an algorithm for automatic parking slot detection as well as an occupancy classification model. Their approach contains several steps: detect cars in subsequent parking lot images with YOLO for approximately five minutes and extract bounding box centers; cluster and eliminate false detection bounding box centers with a clustering algorithm, and from cluster centers, detect bounding boxes of parking spaces; and perform the classification for detected parking spaces. Duong et al. [19] introduced an object detector for parking occupancy detection, OcpDet, based on RetinaNet, with its backbone, ResNet, replaced by MobileNet. They mainly emphasize scalability and reliability. Martynova et al. [20] created a new seasonal dataset for parking lot occupancy detection and developed a custom model based on EfficientNet-B0 for parking occupancy detection.

3. Paragraph 1 in Section 3 is corrected as below:

CNRPark-EXT and PKLot datasets were used in our experiments as the source of data. Table 2 shows the CNRPark-EXT and PKLot dataset features.

4. The Paragraphs in Section 3.1 are corrected as below:

Amato et al. [7] developed the CNRPark-EXT dataset by extending the CNRPark dataset [21]. CNRPark-EXT is a comprehensive dataset designed for visual occupancy detection in parking lots. Figure 7 shows some examples from different camera perspectives and environmental conditions.

CNRPark-EXT contains approximately 150,000 labeled images (patches) representing both vacant and occupied parking spaces. The dataset is built on a parking lot with 164 parking spaces. It extends the original CNRPark dataset, which consisted of 12,000 images collected from two cameras during different days in July 2015. CNRPark-EXT is an additional subset, collected from November 2015 to February 2016, that significantly expands the dataset. It includes images captured by nine cameras with varying perspectives and angles of view. CNRPark-EXT captures diverse scenarios, including different light conditions, partial occlusions (due to obstacles like trees, lampposts, and other cars), and partial or global shadows on cars. The cameras in CNRPark-EXT cover a wide range of views, capturing parking spaces from different angles. The dataset provides a glimpse into the fields of view of the nine available cameras.

5. Paragraph 1 in Section 3.2 is corrected as below:

The PKLot dataset is a robust collection designed specifically for parking lot classification developed by Almeida et al. [8]. The PKLot dataset comprises 12,417 images of parking lots and an impressive 695,899 images of segmented parking spaces. The dataset incorporates images captured under various weather conditions, including sunny, cloudy, and rainy days, ensuring the model’s robustness to weather variations. Images were collected at different times of the day, including diverse lighting conditions that a real-world parking detection system would encounter. The dataset was acquired from the parking lots of two Brazilian universities: the Federal University of Parana (UFPR) and the Pontificial Catholic University of Parana (PUCPR), both located in Curitiba, Brazil. Investigations have revealed that UFPR04 presents a slightly greater challenge than the other two subsets, UFPR05 and PUCPR; this is because this subset contains images with different obstacles and ground patterns. The dataset includes both occupied and empty parking spaces, allowing for comprehensive classification tasks. The dataset contains images of parking lots with delimited spaces, both occupied and empty. Figure 8 shows some examples from all three different camera points in different weather conditions.

6. The mentions of “Figure 12” should be “Figure 9”, “Figure 13” should be “Figure 10”, “Figure 14” should be “Figure 11”, “Figure 15” should be “Figure 12”, “Figure 16” should be “Figure 13”, and “Figure 17” should be “Figure 14”.

7. In Paragraph 19 in Section 5, the content “while VGGNet-F, proposed by Valipour et al. [27], needed about 86MB” is removed.

## References Correction

References [11–17,19–23,26–36,42] are removed. Seven new references [11–13,17,25–27] are added. With this correction, the order of some references has been adjusted accordingly. The correct References section appears below:

1. Almeida, P.R.; Alaves, J.H.; Parpinelli, R.S.; Barddal, J.P. A Systematic Review on Computer Vision-Based Parking Lot Management Applied on Public Datasets. *Expert Syst. Appl.* **2022**, *198*, 116731.
2. Howard, A.; Sandler, M.; Chu, G.; Chen, L.-C.; Chen, B.; Tan, M.; Wang, W.; Zhu, Y.; Pang, R.; Vasudevan, V.; et al. Searching for MobileNetV3. In Proceedings of the IEEE/CVF International Conference on Computer Vision, Seoul, Republic of Korea, 27 October–2 November 2019; pp. 1314–1324.
3. Zhang, Y.; Chen, X. Lightweight Semantic Segmentation Algorithm Based on MobileNetV3 Network. In Proceedings of the 2020 International Conference on Intelligent Computing, Automation and Systems (ICICAS), Chongqing, China, 11–13 December 2020; pp. 429–433.
4. Hu, J.; Shen, L.; Albaine, S.; Sun, G.; Wu, E. Squeeze-and-Excitation Networks. In Proceedings of the 2018 IEEE/CVF Conference on Computer Vision and Pattern Recognition, Salt Lake City, UT, USA, 18–23 June 2018; pp. 7132–7141.
5. Jia, L.; Wang, Y.; Zang, Y.; Li, Q.; Leng, H.; Xiao, Z.; Long, W.; Jiang, L. MobileNetV3 with CBAM for Bamboo Stick Counting. *IEEE Access* **2022**, *10*, 53963–53971.
6. Haase, D.; Amthor, M. Rethinking Depthwise Separable Convolutions: How Intra-Kernel Correlations Lead to Improved MobileNets. In Proceedings of the 2020 IEEE/CVF Conference on Computer Vision and Pattern Recognition (CVPR), Seattle, WA, USA, 13–19 June 2020; pp. 14588–14597.
7. Amato, G.; Carrara, F.; Falchi, F.; Gennaro, C.; Meghini, C.; Vairo, C. Deep Learning for Decentralized Parking Lot Occupancy Detection. *Expert Syst. Appl.* **2017**, *72*, 327–334.
8. Almeida, P.R.; Oliveira, L.S.; Britto, A.S., Jr.; Silva, E.J., Jr.; Koerich, A.L. PKLot—A Robust Dataset for Parking Lot Classification. *Expert Syst. Appl.* **2015**, *42*, 4937–4949.
9. Howard, A.; Zhu, M.; Chen, B.; Kalenichenko, M.; Wang, W.; Weyand, T.; Andreetto, M.; Adam, H. MobileNets: Efficient Convolutional Neural Networks for Mobile Vision Applications. *arXiv* **2017**, arXiv:1704.14861.
10. Sandler, M.; Howard, A.; Zhu, M.; Zhmoginov, A.; Chen, L.-C. MobilenetV2: Inverted Residuals and Linear Bottlenecks. In Proceedings of the 2018 IEEE/CVF Conference on Computer Vision and Pattern Recognition (CVPR), Salt Lake City, UT, USA, 18–23 June 2018; pp. 4510–4520.
11. Al-Kharusi, H.; Al-Bahadly, I. Intelligent parking management system based on image processing. *World J. Eng. Technol.* **2014**, *2*, 55–67.
12. Ahrnbom, M.; Astrom, K.; Nilsson, M. Fast classification of empty and occupied parking spaces using integral channel features. In Proceedings of the 2016 IEEE Conference on Computer Vision and Pattern Recognition Workshops (CVPRW), Las Vegas, NV, USA, 26 June–1 July 2016; pp. 1609–1615.
13. Razavian, A.S.; Azizpour, H.; Sullivan, J.; Carlsson, S. CNN features off-the shelf: An astounding baseline for recognition. In Proceedings of the 2014 IEEE Conference on Computer Vision and Pattern Recognition Workshops (CVPRW), Columbus, OH, USA, 23–28 June 2014; pp. 512–519.
14. Krizhevsky, A.; Sutskever, I.; Hinton, G.E. Imagenet Classification with Deep Convolutional Neural Networks. In *Advances in Neural Information Processing Systems*; MIT Press: Cambridge, MA, USA, 2012; pp. 1097–1105.
15. Nguyen, T.; Tran, T.; Mai, T.; Le, H.; Le, C.; Pham, D.; Phung, K.H. An Adaptive Vision-based Outdoor Car Parking Lot Monitoring System. In Proceedings of the 2020 IEEE Eighth International Conference on Communications and Electronics (ICCE), Phu Quoc Island, Vietnam, 13–15 January 2021; pp. 445–450.
16. Nurullayev, S.; Lee, S.-W. Generalized Parking Occupancy Analysis Based on Dilated Convolutional Neural Network. *Sensors* **2019**, *19*, 277.
17. Xiao, A.; Doshi, D.; Wang, L.; Gorantla, H.; Heitzmann, T.; Groth, P. Parking Spot Classification based on surround view camera system. *arXiv* **2023**, arXiv:2310.12997.
18. Grbić, R.; Koch, B. Automatic Vision-Based Parking Slot Detection and OCCUPANCY classification. *Expert Syst. Appl.* **2023**, *225*, 120147.
19. Duong, T.L.; Le, V.D.; Bui, T.C.; To, H.T. Towards an Error-free Deep Occupancy Detector for Smart Camera Parking System. In Proceedings of the European Conference on Computer Vision, Tel Aviv, Israel, 23–27 October 2020; Springer: Cham, Switzerland, 2022; pp. 163–178.
20. Martynova, A.; Kuznetsov, M.; Porvatov, V.; Tishin, V.; Kuznetsov, A.; Semenova, N.; Kuznetsova, K. Revising Deep Learning Methods in Parking Lot Occupancy Detection. *arXiv* **2023**, arXiv:2306.04288.
21. Amato, G.; Carrara, F.; Falchi, F.; Gennaro, C.; Vairo, C. Car Parking Occupancy Detection using Smart Camera Networks and Deep Learning. In Proceedings of the 2016 IEEE Symposium on Computers and Communication (ISCC), Messina, Italy, 27–30 June 2016; Institute of Electrical and Electronics Engineers Inc.: New York, NY, USA, 2016; Volume 2016, pp. 1212–1217.
22. Selvaraju, R.R.; Cogswell, M.; Das, A.; Vedantam, R.; Parikh, D.; Batra, D. Grad-CAM: Visual Explanations from Deep Networks via Gradient-Based Localization. In Proceedings of the 2017 IEEE International Conference on Computer Vision (ICCV), Venice, Italy, 22–29 October 2017; pp. 618–626.
23. Nguyen, A.; Yosinski, J.; Clune, J. Understanding Neural Networks via Feature Visualization: A Survey. In *Explainable AI: Interpreting, Explaining and Visualizing Deep Learning*; Samek, W., Montavon, G., Vedaldi, A., Hansen, L., Müller, K.R., Eds.; Lecture Notes in Computer Science; Springer: Berlin/Heidelberg, Germany, 2019; Volume 11700, pp. 55–76.
24. Simonyan, K.; Zisserman, A. Very Deep Convolutional Networks for Large-Scale Image Recognition. *arXiv* **2014**, arXiv:1409.1556.
25. Chollet, F. Xception: Deep Learning with Depthwise Separable Convolutions. In Proceedings of the 2017 IEEE Conference on Computer Vision and Pattern Recognition (CVPR), Honolulu, HI, USA, 21–26 July 2017; pp. 1251–1258.
26. Szegedy, C.; Vanhoucke, V.; Ioffe, S.; Shlens, J.; Wojna, Z. Rethinking the Inception Architecture for Computer Vision. In Proceedings of the 2016 IEEE Conference on Computer Vision and Pattern Recognition (CVPR), Las Vegas, NV, USA, 26 June–1 July 2016; pp. 2818–2826.
27. He, K.; Zhang, X.; Ren, S.; Sun, J. Deep Residual Learning for Image Recognition. In Proceedings of the IEEE Conference on Computer Vision and Pattern Recognition (CVPR), Las Vegas, NV, USA, 26 June–1 July 2016; pp. 770–778.
28. Satyanath, G.; Sahoo, J.K.; Roul, R.K. Smart Parking Space Detection under Hazy Conditions using Convolutional Neural Networks: A Novel Approach. *Multimed. Tools Appl.* **2023**, *82*, 15415–15438.

The authors apologize for any convenience caused and state that the scientific conclusions are unaffected. This correction was approved by the Academic Editor. The original article has been updated.

## Figures and Tables

**Figure 6:**
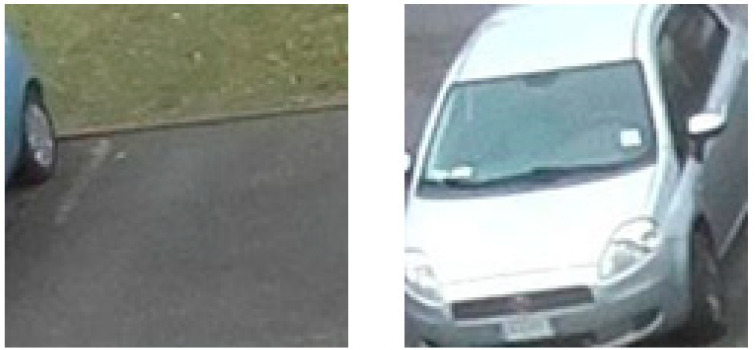
Empty and busy parking spaces.

**Figure 7:**
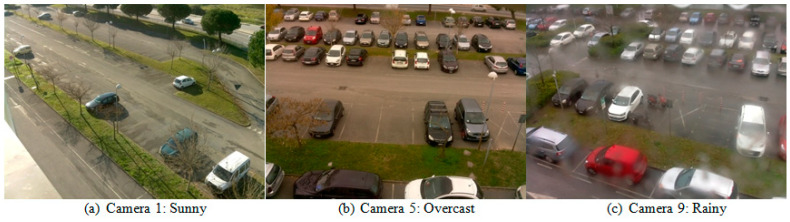
CNRPark-EXT dataset samples: (**a**–**c**) taken in 3 weather conditions.

**Figure 8:**
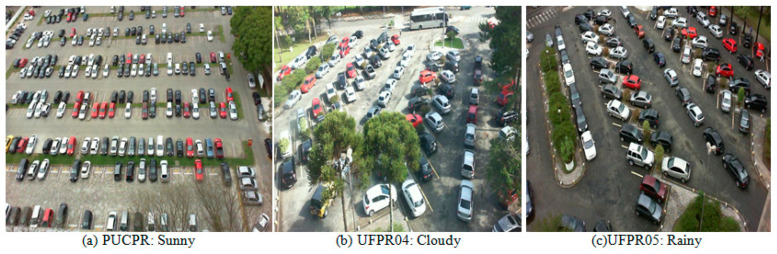
PKLot dataset samples. (**a**–**c**) show examples of different parking lots and weather conditions.
